# Development and validation of nomogram based on a novel platelet index score to predict prognosis in patients with renal cell carcinoma

**DOI:** 10.7150/jca.60268

**Published:** 2021-08-28

**Authors:** Ruotao Xiao, Yanchun Qin, Lei Liu, Zhigang Chen, Bin Yang, Chuxiao Xu, Wei He, Cheng Liu, Lulin Ma

**Affiliations:** Department of Urology, Peking University Third Hospital, Beijing, China

**Keywords:** nomogram, platelet count, platelet distribution width, platelet index score, renal cell carcinoma, progression-free survival

## Abstract

**Purpose:** This study aims to develop and validate a nomogram based on a novel platelet index score (PIS) to predict prognosis in patients with renal cell carcinoma (RCC).

**Patients and methods:** We retrospectively analyzed the data of 759 consecutive patients with RCC. The Kaplan-Meier curves were performed to analyze the platelet parameters and PIS was established. The patients were randomly divided into training (N=456, 60%) and validation cohorts (N=303, 40%). The nomogram was created based on the factors determined by multivariable Cox proportional hazard regression of the training cohort. We assessed the discrimination and calibration of our nomogram in both training and validation cohorts. And then the nomogram was compared with other reported models.

**Results:** High platelet count (PLT>285×10^9^/L) and low platelet distribution width (PDW≤10.95fL) were associated with shorter progression-free survival (PFS). Thus, PLT and PDW were incorporated in a novel score system called PIS. On multivariable analysis of training cohort, PIS, American Joint Committee on Cancer (AJCC) stage, and sarcomatoid differentiation were independent prognostic factors, which were all selected into the nomogram. The nomogram exhibited good discrimination in both training (C-index: 0.835) and validation cohorts (C-index: 0.883). The calibration curves also showed good agreement between prediction and observation in both cohorts. The C-index of the nomogram (C-index: 0.810~0.902) for predicting 2-year, 3-year, and 4-year PFS were significantly higher than Leibovich (C-index: 0.772~0.813), SSIGN (C-index: 0.775~0.876), Cindolo (C-index: 0.642~0.798), Yaycioglu (C-index: 0.648~0.804), MSKCC (C-index: 0.761~0.862), Karakiewicz (C-index: 0.747~0.851), and AJCC stage models (C-index: 0.759~0.864).

**Conclusion:** The nomogram based on a novel PIS could offer better risk stratification in patients with RCC.

## Introduction

Renal cell carcinoma (RCC) is the third most common genitourinary worldwide, accounting for 3%~5% of the malignancies in adults 1. Localize RCC can be cured by surgery, with a favorable 5-year overall survival of 71%~88% 2. However, even with a relatively good prognosis, approximately 28% of patients will experience recurrence after curative surgery 3. Nowadays several prognostic models like SSIGN, Leibovich, Cindolo, Yaycioglu, MSKCC, and Karakiewicz models have been used in predicting survival for patients with RCC. The reported concordance index (C-index) of the above models were range from 0.67 to 0.84^3^-^8^. However, in a prospective external validation cohort, the predictive ability was decreased with a C-index range from 0.587 to 0.69^9^. Thus, it is still worthy for us to further explore the potential prognostic factors and develop more accurate predictive models for RCC patients.

Platelets have been recognized as the key factor in hemostasis and wound healing. Recent evidence demonstrated that cancer cells can activate platelets and induce aggregation, which in turn contribute to cancer genesis and metastasis 10. Platelet count (PLT) is the most notable index that can be affected by the equilibrium between production and consumption. High PLT has been reported as an unfavorable prognostic factor in many malignancies including RCC 11-14. Recently, mean platelet volume (MPV) and platelet distribution width (PDW), which reflect the size and uniformity of platelets, have shown their prognostic value in thyroid, lung, gastric, and breast cancers 15-18.

To our knowledge, there are few studies that explore the prognostic value of MPV and PDW in RCC populations. Besides, previous models contain similar variables like ECOG-PS, TNM stage, Fuhrman grade, symptom, necrosis, tumor size, et al. 3-8. However, none of these prognostic models have included any platelet parameters, except the International Metastatic Renal Cell Carcinoma Database Consortium model (IMDC) which was included PLT to predict survival of metastatic RCC^19^. Thus, our study aims were as follows: (1) To explore the prognostic value of PLT, MPV, and PDW in RCC patients and develop a novel platelet index score (PIS); (2) To develop and validate nomogram based on the PIS in our training and validation cohorts; (3) To compare the predictive ability of our nomogram with other reported prognostic models.

## Material and methods

### Patients selection

**Figure 1:**
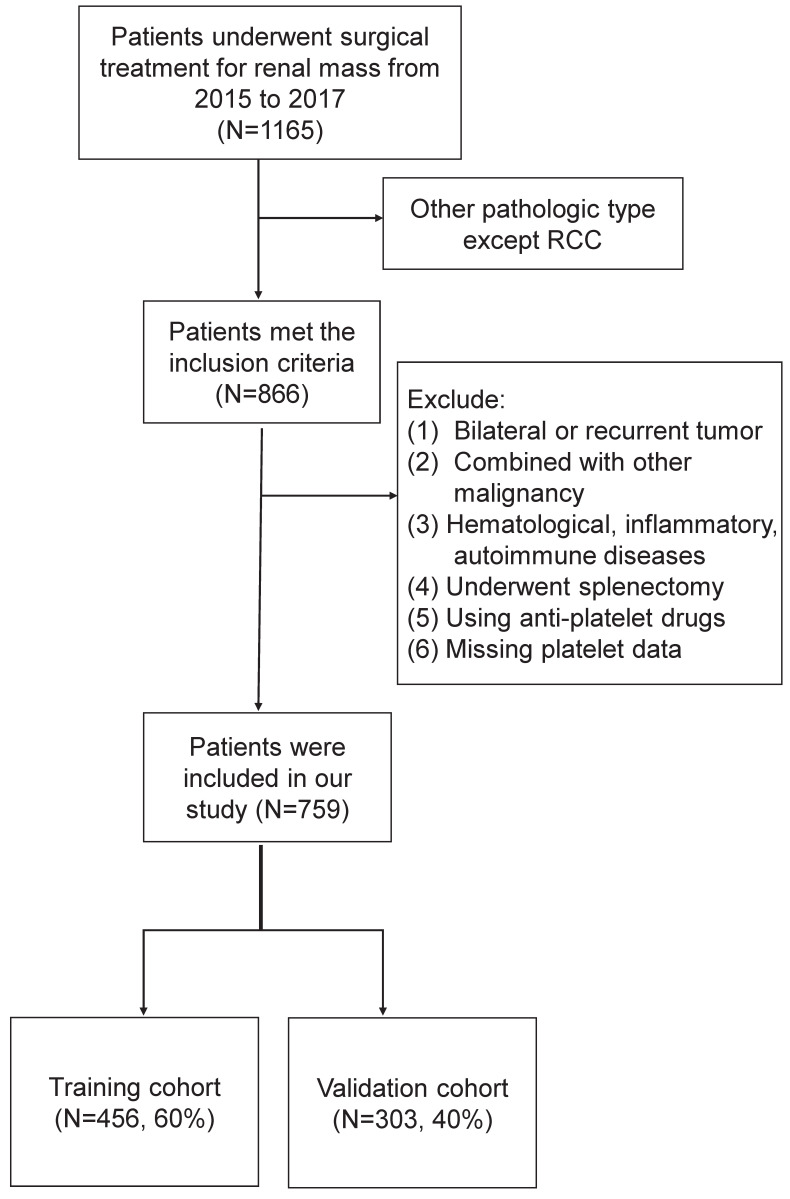
The flow chart of our study cohort.

The clinicopathologic data of renal mass admitted to Peking University Third Hospital from January 2015 to December 2017 were retrospectively analyzed. Inclusion criteria were patients who underwent nephrectomy in our department and pathologically confirmed RCC. Exclusion criteria were as follows: (1) recurrent or bilateral RCC; (2) combined with other malignancies; (3) those with the hematological disease, inflammatory disease, autoimmune disease; (4) those who underwent splenectomy in the past history; (5) those using antiplatelet drugs withing a week before blood collection; (6) missing platelet data. The flow chart was shown in Figure 1. Finally, the eligible study cohort consisted of 759 consecutive RCC patients were further analyzed.

### Data collection

All patients underwent a blood tests and image examinations. Blood collection was performed within a week before surgery. The whole blood sample was collected in EDTA-containing tubes and processed within an hour after collection. All patients underwent chest X-ray, B-mode ultrasonography, abdominal computed tomography (CT), and/or magnetic resonance imaging (MRI) before surgery. Position emission CT, chest CT, cranial MRI, and bone scans were performed when suspicious distant metastasis. Surgical type and approach were determined by the surgeon' preference based on the current guidelines. All tumor specimen was evaluated by two experienced pathologists. Patients with distant metastasis were treated using tyrosine kinase (TKI) inhibitors as the first-line adjuvant target therapy.

Clinicopathologic variables were collected, including age, sex, body mass index (BMI), symptom at presentation, comorbidities, surgical type, surgical approach, surgical time, interoperative blood loss, tumor size, tumor side, tumor stage, histologic subtype, nuclear grade, necrosis, sarcomatoid and rheumatoid differentiation as well as platelet parameters including PLT, MPV, and PDW through electronic medical records of Peking University Third Hospital.

Surgical type, approach, surgical time, and interoperative blood loss were determined based on surgical records. Tumor size, tumor side were evaluated according to the preoperative CT/MRI image report. The tumor stage was evaluated according to both preoperative image and postoperative pathology report, and defined based on the 2016 WHO TNM classification 20. Nuclear grades were defined according to the 2016 WHO/ISUP grading system 20.

### Follow up

Patients were recommended to follow up regularly at our institution for every 3 months during the first year, every 6 months for the following year, and then annually. Blood tests and chest x-ray were performed at each follow-up while abdominal CT scan was done annually. Follow-up data were collected through medical records or telephone calls. Disease progression was defined as any evidence of recurrence or metastasis, or tumor progress in an already existed metastatic site. Progression-free survival (PFS) was defined as the time from the date of surgery to disease progression.

### Statistical analysis

Normally distributed continuous variable was reported as mean ± standard deviations. Non-normally distributed continuous variables were reported as medians and interquartile range (IQR). Categorical variables using the frequency count and percentage. The analysis of variance (ANOVA) test or Kruskal-Wallis test were applied to compare continuous variables. The Chi-square test or Fisher exact test was applied to compare categorical variables. The receiver operating characteristic curve (ROC) was used to determine the optimal cut-off value of PLT, MPV, PDW according to survival outcome. The Kaplan-Meier method was performed to analyze the platelet parameters, and the difference between groups was compared using the log-rank test.

To develop a reliable and well-calibrated nomogram, patients were randomly divided into training cohorts (N=456, 60%) and validation cohorts (N=303, 40%). Univariable and multivariable Cox proportional hazard regression were performed to identify the independent prognostic factors in the training cohort. Selected independent factors were incorporated in the nomogram to predict the probability of 2-year, 3-year, and 4-year PFS. The discrimination of the nomogram was measured by C-index, which ranges from 0.5 (no predictive power) to 1 (perfect prediction) 21. Calibration was evaluated using calibration curves, which assessed between the observed outcome probabilities and the nomogram predicted probabilities with a bootstrapped resample of 1000 times. The total points of each patient in the validation cohort were calculated according to the nomogram, then Cox regression was performed using the total points as a factor, and finally, the C-index and calibration curve was derived based on the regression analysis. Time-dependent ROC curves and areas under the curves (AUCs) at 2-year, 3-year, and 4-year were generated to assess prognostic accuracy of the nomogram in training and validation cohorts 22.

Improvement in the predictive accuracy was assessed by calculating the integrated discrimination improvement (IDI) and the net reclassification improvement (NRI) 23. Higher IDI and NRI indicated greater risk discrimination and improved classification. Furthermore, the linear predictor was calculated as the sum of the product of regression coefficients and variables reported by other models, and C-index was calculated according to the linear predictor and then compared with our nomogram. Nomogram establishment and calibration were conducted by R software (Version 4.0.3) using the “rms” package, and other statistical analysis was performed with SPSS (Version 26, IBM, Armonk, NY) and GraphPad Prism (Version 8, GraphPad Software Inc., San Diego, CA, USA). All tests were on two sided, and P<0.05 was considered statistically significant.

## Results

### Study cohort and platelet index score establishment

**Figure 2:**
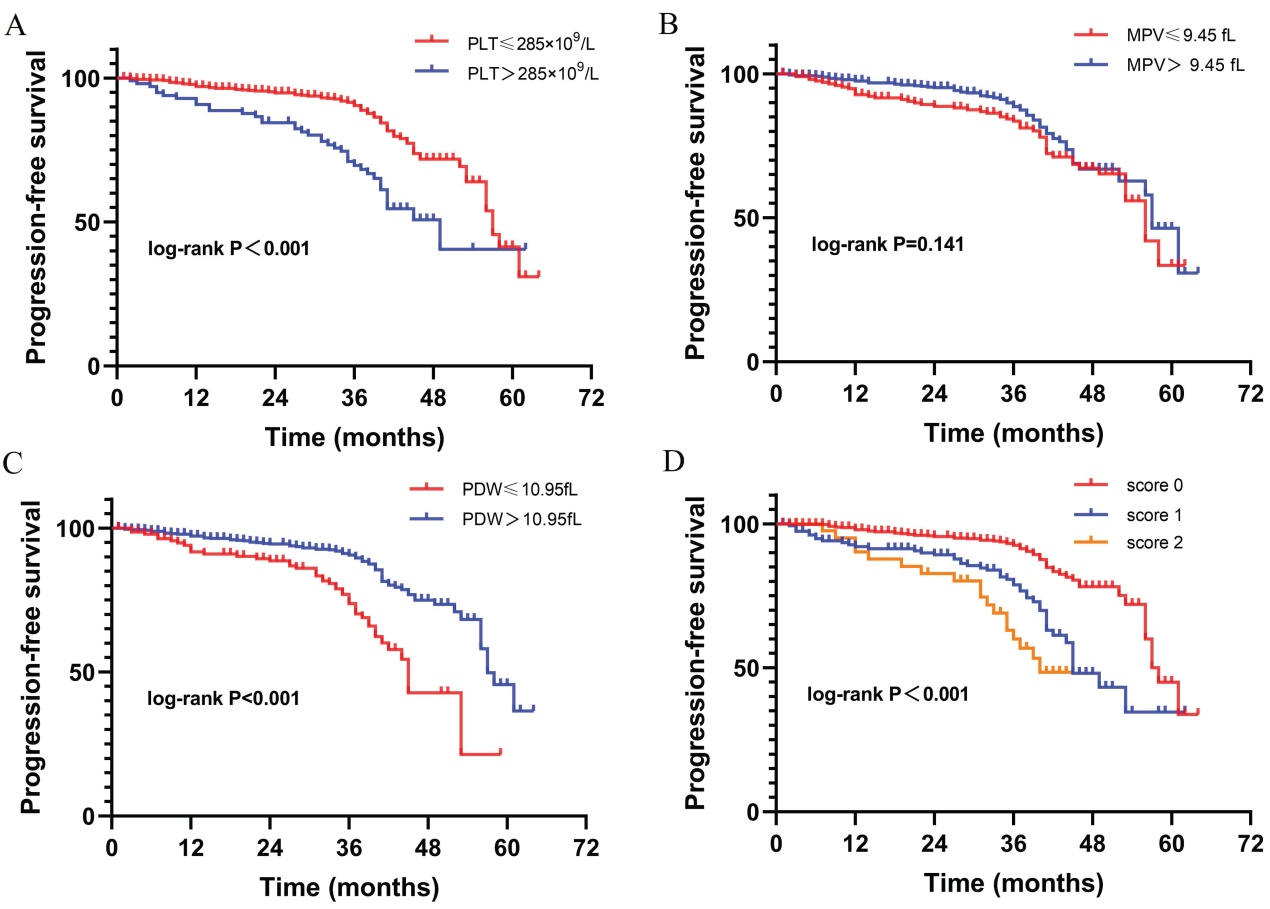
The Kaplan-Meier curves correlated with progression-free survival for platelet parameters. (A) PLT; (B) MPV; (C) PDW; (D) PIS.

Included in the analysis were 759 patients (**Table 1**). The characteristics of training and validation cohorts were similar. The detailed profile of PLT, MPV, and PDW were shown in **Supplement 1**. The range of PLT, MPV, and PDW was 57~689×10^9^/L, 6.2~20.5fL, and 7.9~23.1fL, respectively. Besides, the mean PLT, MPV, and PDW was 223.45±69.31×10^9^/L, 10.01±1.4fL, and 13.57±2.78fL respectively. Of 665 (87.6%) patients were followed up with the median time of 36 months (IQR: 31~41). Of 126 patients (16.6%) reported disease progression with a median PFS of 57 months (IQR: 43~ Not reached). The optimal cut-off values of PLT, MPV, and PDW were 285×10^9^/L, 9.45fL, 10.95fL respectively, with the maximal Youden index on ROC analysis (**Supplement 2**). Then dichotomization of PLT, MPV, and PDW was performed according to the cut-off value. Kaplan-Meier curves show high PLT (P<0.001) and low PDW (P<0.001) were significantly associated with shorter PFS. While MPV (P=0.141) was not significantly associated with PFS in our study cohort (**Fig. 2**).

To further investigate the prognostic value of platelet parameters in RCC, we established a platelet index score (PIS) by combining PLT and PDW. The PIS were defined as follows: (1) Score 0: patients with low PLT (≤285×10^9^/L) and high PDW (>10.95fL); (2) Score 1: patients with either high PLT (>285×10^9^/L) or low PDW (≤10.95fL); (3) Score 2: patients with high PLT (>285×10^9^/L) and low PDW (≤10.95fL). In our study cohort, the cases of PIS score 0, 1, 2 were 531 (70%), 178 (23.5%), 50 (6.6%) respectively. The Kaplan-Meier curve shows patients with score 0 have significantly longer PFS than those with score 1 and score 2 (P<0.001, **Fig. 2**). Besides, patients with higher PIS associated with lower BMI (P=0.002), more symptom at presentation (P=0.035), larger tumor size (P<0.001), advanced AJCC stage (P<0.001) and nuclear grade (P=0.001), more necrosis (P=0.004), sarcomatoid differentiation (P=0.043), and rheumatoid differentiation (P=0.001) (**Table 2**).

### Nomogram development and validation

**Figure 3:**
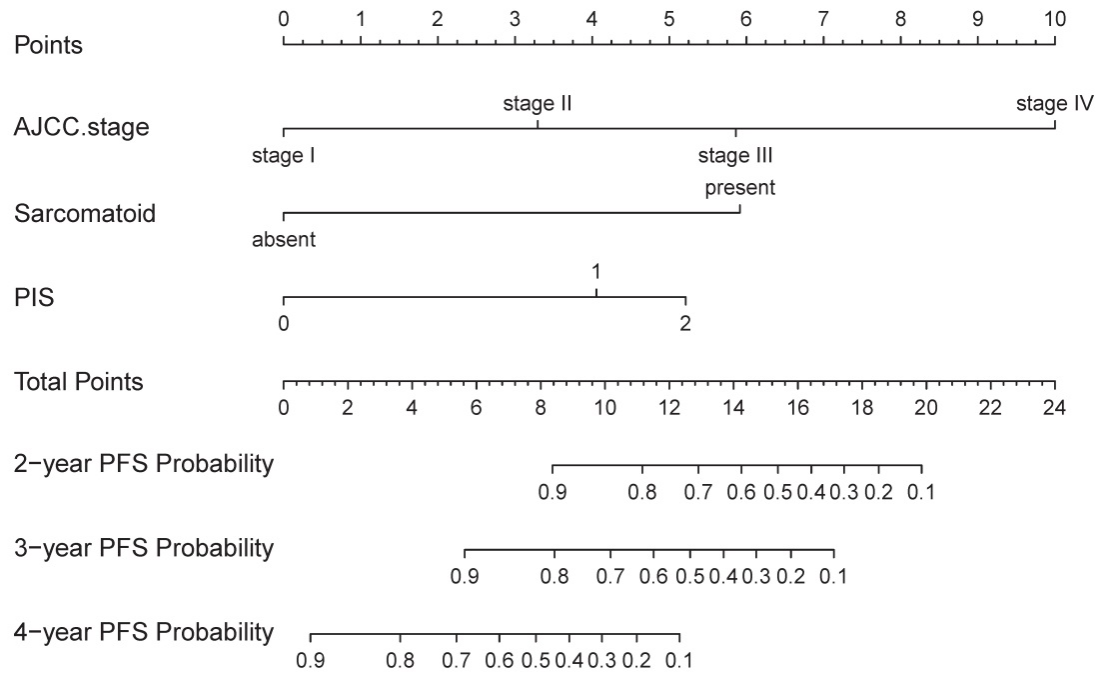
The nomogram was developed in the training cohort, with AJCC Stage, Sarcomatoid differentiation, PIS.

**Figure 4:**
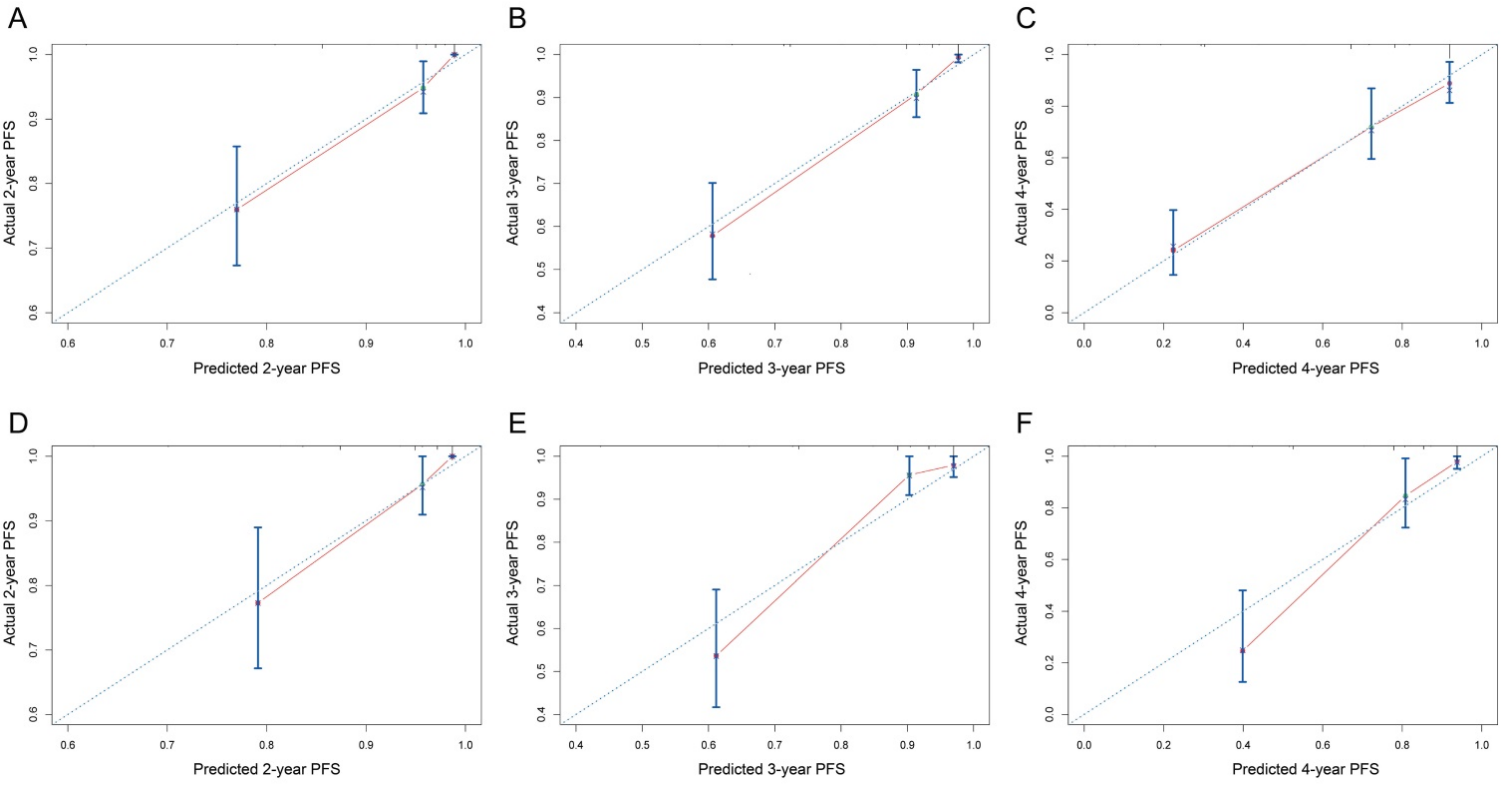
The calibration curves of nomogram to predict progression-free survival in training cohort and validation cohort. (A-C) 2-year, 3-year, and 4-year calibration curves for training cohort; (D-F) 2-year, 3-year, and 4-year calibration curves for validation cohort.

**Figure 5:**
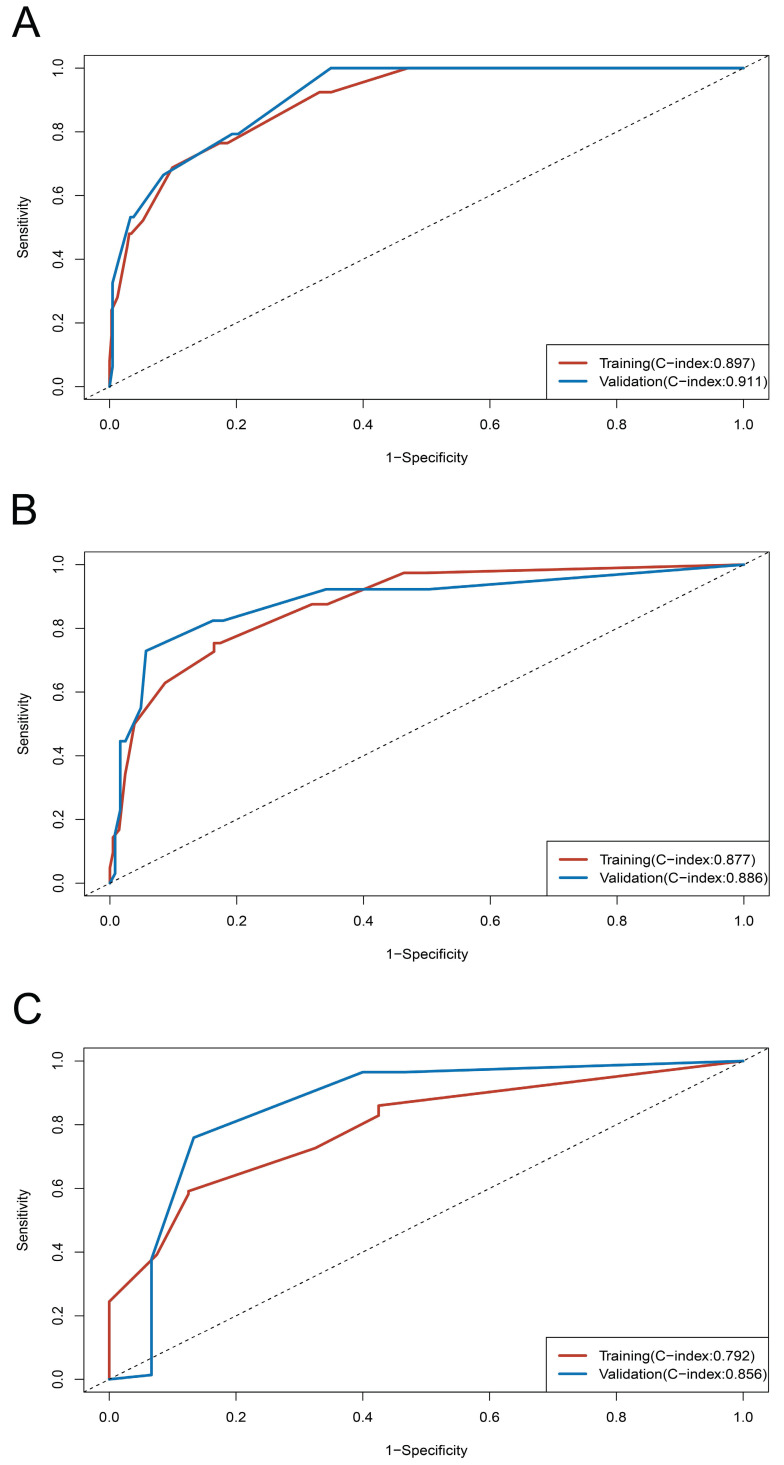
Time-dependent ROC curves and C-index of training and validation cohorts. (A) ROC curve at 2-year; (B) ROC curve at 3-year; (C) ROC curve at 4-year.

On univariable and multivariable analysis in the training cohort, higher PIS (HR: 2.098 for score 1, 2.775 for score 2, P<0.001), higher AJCC stage (HR: 2.746 for II, 5.352 for III, 13.662 for IV, P<0.001), and sarcomatoid differentiation (HR: 2.032, P=0.030) were independent factors predict shorter PFS (**Table 3**). Thus, we established the nomogram by incorporating all the independent factors to predict 2-year, 3-year, and 4-year PFS (**Fig. 3**). The C-index was 0.835 for the training cohort and the calibration curves for the probability of 2-year, 3-year, and 4-year PFS showed an optimal agreement with the prediction by nomogram and actual observation (**Fig. 4**). In the validation cohort, the C-index was 0.883 and calibration curves also showed good agreement between prediction and observation (**Fig. 4**). To further compare the discrimination of the nomogram in training and validation cohorts, the 2-year, 3-year, and 4-year of time-dependent ROC curves were showed in **Figure 5**, which suggested good discrimination in both training and validation cohorts.

The NRI and IDI were performed to show the improvement of the nomogram compared to the model without PIS. The NRI were 0.284 (P<0.001), 0.384 (P<0.001), and 0.307 (P=0.02) for 2-year, 3-year, and 4-year PFS respectively, which significantly improved compared to the model without PIS. The IDI were 0.056 (P=0.07), 0.076 (P<0.001), and 0.08 (P=0.03) for 2-year, 3-year, and 4-year PFS respectively, which also significantly improved compared to the model without PIS.

### Comparison of our nomogram and other models

Of our study cohort, the C-index of our nomogram was 0.902, 0.881, 0.810 respectively in predicting 2-year, 3-year, and 4-year PFS (**Table 4**). Models of SSIGN 4, Leibovich 3, Cindolo 6, Yaycioglu 5, MSKCC 7, Karakiewicz 8, and conventional AJCC staging system 20 were externally validated in our study cohort. The SSIGN performed the best (C-index: 0.775~0.876) and Cindolo performed the worst (C-index: 0.642~0.798). Besides, our nomogram displayed a better accuracy than Leibovich, SSIGN model, Cindolo, Yaycioglu, MSKCC, Karakiewicz, and AJCC stage models.

## Discussion

The ability to predict oncologic outcomes in RCC patients is essentially significant to clinicians. Platelets parameters have been explored as a promising biomarker in predicting oncologic outcomes in cancers. In this study we analyzed a consecutive 759 RCC patients and reported several noteworthy findings. Firstly, high PLT and low PDW were associated with shorter PFS in our study group. Then we established a novel score system named PIS by combining PLT and PDW, which have shown their significant correlation with disease progression. Secondly, we created a nomogram based on the PIS and other independent factors to predict PFS, which exhibited good discrimination and calibration in the training and validation cohort. Thirdly, our nomogram displayed better accuracy in predicting 2-year, 3-year, and 4-year PFS than other reported models and conventional AJCC staging system.

Notably, several studies have explored that high PLT was related to worse prognosis in RCC populations, which consistent with our findings 14, 24. Recent evidence also showed increased PLT had a lower response rate to tyrosine kinase inhibitors therapy and shorter overall survival in metastatic RCC 25. Thus, PLT was included in the IMDC model to predict prognosis for metastatic RCC patients 19. However, the conflicting results were also reported that PLT was not an independent factor predicting prognosis after adjusting some known pathologic factors, which suggested we should focus more on other platelet parameters that can better reflect the function rather than platelet count alone 26, 27.

Preclinical studies have found cancer cells can activate platelet through a wide variety of crosstalk, eventually caused tumor genesis and metastasis 8. MPV and PDW can serve as indicators reflecting the activation of platelets 28, 29. Clinical studies have found a strong correlation with diagnosis and prognosis in several malignancies 15-18. Seles M et al. 30 have found MPV represented a highly significant predictor of recurrence and cancer-specific death in patients with RCC. Although our study failed to show the prognostic value of MPV, the PDW was another novel biomarker in predicting prognosis for RCC patients, which has not been investigated before. Several studies have shown high PDW was associated with poor survival in hepatocellular, breast cancers as well as skull base chordoma 31-33. However, low PDW has also been reported that correlated with worse survival in endometrial, esophageal, gastric cancers 34-36, which consistent with our study result that low PDW was related to shorter PFS in RCC patients. The reason for the conflicting results was unclear. We speculate that within the tumor microenvironment, tumor cells can trigger activated platelet release granules that contain platelet-derived growth factor (PDGF), vascular endothelial growth factor (VEGF), and transforming growth factor β (TGF-β), which lead to decreased in size and increased heterogeneity of platelet at an early stage of tumor 10. But as tumor growth and tumor burden increased, more and more platelets were affected by cancer cells, and eventually, the heterogeneity was decreased. However, the underline mechanism remains to be elucidated.

Furthermore, we created a novel score system called PIS by combining PLT and PDW and found that high PIS correlated with shorter PFS in RCC. Chen H et al. 36 was the first to established a similar platelet index score by combining PLT, MPV, and PDW, which showed its strong correlation with recurrent-free survival and overall survival in endometrial cancer. It is worth noting that in RCC patients, conventional prognostic models, except the IMDC model, were not included any platelet parameters 3-8, 19. Since our study result has shown the prognostic value of PIS, we created a nomogram based on PIS to predict PFS in patients with RCC. The nomogram performed well in the prediction of RCC patients by its reported C-index (0.835 and 0.883 for the training and validation cohort) and calibration curve. Although the external validation of other models has also shown its good discrimination with C-index range from 0.642 to 0.895, the nomogram is more accurate to predict PFS than the models like Leibovich, SSIGN, Cindolo, MSKCC, Karakiewicz, and Yaycioglu models as well as AJCC staging system. Besides, our nomogram only included three features, which was simpler than other models. More importantly, our findings not only raise significant concerns regarding the prognostic value of preoperative platelet parameters in patients with RCC but also predict prognosis for patients with RCC in a more accurate way by combining platelet parameters with conventional prognostic factors.

Several limitations may apply to our findings. We cannot eliminate the inherent bias owing to the retrospective nature of our study. As we know the preoperative drugs, inflammation, and hematologic disease may affect platelet function and parameters. But we could only achieve this information from the electronic medical records, which may not reflect the real state of an individual. Besides, although our study demonstrated that the PDW and PLT were associated with prognosis, the cut-off value of these parameters varied between different studies. Finally, the nomogram was only developed and validated in a retrospective single-center population. Thus, future studies are needed to externally validate the proposed nomogram in a multicenter, prospective, and large cohort.

## Conclusion

In conclusion, we found high PLT, low PDW, and high PIS were associated with shorter PFS in patients with RCC. Then we established a nomogram based on the PIS for predicting PFS in patients with RCC. The nomogram could offer better risk stratification than other reported prognostic models in patients with RCC.

### Ethical approval

The study was conducted in accordance with the Declaration of Helsinki and was approved by the Peking University Third Hospital Medical Science Research Ethics Committee. Because of the retrospective nature of the study, patient consent for inclusion was waived.

## Supplementary Material

Supplementary figures.

## Figures and Tables

**Table 1:** Demographics and clinicopathologic characteristics of patients with renal cell carcinoma

Variable	Study cohort (N=759)	Training cohort (N=456)	Validation cohort (N=303)	P
Age, years	56.47±12.03	56.56±11.98	56.33±12.12	0.793
Sex				
Male	523 (68.9)	312 (68.4)	211 (69.6)	0.749
Female	236 (31.1)	144 (31.6)	92 (30.4)	
Symptom at presentation	176 (23.2)	104 (22.8)	72 (23.8)	0.792
BMI, kg/m^2^	25.15±3.41	25.21±3.55	25.06±3.19	0.552
Comorbidities				
Hypertension	300 (39.5)	190 (41.7)	110 (36.3)	0.150
Coronary heart disease	63 (8.3)	37 (8.1)	26 (8.6)	0.893
Diabetes mellitus	117 (15.4)	68 (14.9)	49 (16.2)	0.608
Surgical type				
NSS	378 (49.8)	223 (48.9)	155 (51.2)	0.554
RN	381 (50.2)	233 (51.1)	148 (48.8)	
Surgical approach				
Open	338 (44.5)	208 (45.6)	130 (42.9)	0.502
Laparoscopic	421 (55.5)	248 (54.4)	173 (57.1)	
Tumor size, cm	4.89±3.01	4.93±3.1	4.83±2.87	0.643
Tumor side				
Left	362 (47.7)	215 (47.1)	147 (48.5)	0.767
Right	397 (52.3)	241 (52.9)	156 (51.5)	
AJCC tumor stage				
I	465 (61.3)	277 (60.7)	188 (62)	0.618
II	24 (3.2)	17 (3.7)	7 (2.3)	
III	206 (27.1)	121 (26.5)	85 (28.1)	
IV	64 (8.4)	41 (9.0)	23 (7.6)	
Histologic subtype				
CCRCC	656 (86.4)	398 (87.3)	258 (85.1)	0.449
Non-CCRCC	103 (13.6)	58 (12.7)	45 (14.9)	
Nuclear grade				
I	90 (11.9)	54 (11.8)	36 (11.9)	0.548
II	442 (58.2)	263 (57.7)	179 (59.1)	
III	142 (18.7)	93 (20.4)	49 (16.2)	
IV	39 (5.1)	21 (4.6)	18 (5.9)	
Unknow	46 (6.1)	25 (5.5)	21 (6.9)	
Necrosis	129 (17)	75 (16.4)	54 (17.8)	0.623
Sarcomatoid differentiation	21 (2.8)	12 (2.6)	9 (3)	0.340
Rheumatoid differentiation	18 (2.4)	11 (2.4)	7 (2.3)	0.609
Platelet parameters				
PLT, ×10^9^/L	223.45±69.31	223.42±72.45	223.49±64.4	0.988
MPV, fL	10.01±1.4	10.06±1.38	9.93±1.44	0.184
PDW, fL	13.57±2.78	13.59±2.85	13.53±2.66	0.747
PIS score				
0	531 (70)	323 (70.8)	208 (68.6)	0.821
1	178 (23.5)	104 (22.8)	74 (24.4)	
2	50 (6.6)	29 (6.4)	21 (6.9)	
Follow-up data				
Progression-free	539 (71)	319 (70)	220 (72.6)	0.571
Progression	126 (16.6)	81 (17.8)	45 (14.9)	
Loss of follow up	94 (12.4)	56 (12.3)	38 (12.5)	

**Abbreviations:** BMI: Body mass index; NSS: Nephron sparing surgery; RN: Radical Nephrectomy; AJCC: American Joint Committee on Cancer; CCRCC: Clear cell renal cell carcinoma; PLT: platelet count; MPV: mean platelet volume; PDW: platelet distribution width; PIS score: platelet index score.

**Table 2:** The correlation between platelet index score and clinicopathologic characteristics.

Variable	PIS			P
	0 (N=531)	1 (N=178)	2 (N=50)	
Age, year	56.39±11.98	57.18±12.50	54.84±10.82	0.459
Gender				
Male	369 (69.5)	119 (66.9)	35 (70)	0.793
Female	162 (30.5)	59 (33.1)	15 (30)	
Symptom at presentation	107 (20.7)	51 (29.7)	18 (39.1)	0.002
BMI, kg/m^2^	25.34±3.26	24.81±3.67	24.26±3.80	0.035
Tumor size, cm	4.61±2.88	5.15±2.91	7.01±3.69	<0.001
AJCC stage				
I~II	379 (71.4)	90 (50.6)	20 (40)	<0.001
III~IV	152 (28.6)	88 (49.4)	30 (60)	
Histologic subtype				
CCRCC	433 (86.6)	147 (89.6)	38 (84.4)	0.513
Non-CCRCC	67 (13.4)	17 (10.4)	7 (15.6)	
Nuclear grade				
I-II	387 (78)	118 (70.2)	27 (56.3)	0.001
III-IV	109 (22)	50 (29.8)	21 (43.8)	
Necrosis	82 (15.4)	30 (16.9)	17 (34)	0.004
Sarcomatoid differentiation	11 (2.1)	6 (3.4)	4 (8)	0.043
Rheumatoid differentiation	6 (1.1)	8 (4.5)	4 (8)	0.001

**Abbreviations:** BMI: Body mass index; AJCC: American Joint Committee on Cancer; CCRCC: Clear cell renal cell carcinoma; PIS score: platelet index score.

**Table 3:** Univariable and multivariable analysis to identify the independent factors in the training cohort

Variable	Univariable		Multivariable	
	HR (95%CL)	P	HR (95%CL)	P
Age, year	1.019 (0.999~1.039)	0.059		
Symptom at presentation	2.132 (1.332~3.414)	0.002		
BMI, kg/m^2^	0.890 (0.833~0.950)	0.001		
AJCC stage				
I	Ref		Ref	
II	2.322 (0.529~10.186)	0.264	2.558 (0.577~11.333)	0.216
III	5.836 (3.185~10.693)	<0.001	5.215 (2.784~9.766)	<0.001
IV	16.763 (15.422~46.052)	<0.001	12.664 (6.298~10.698)	<0.001
Histologic subtype (CCRCC vs Non-CCRCC)	1.107 (0.501~2.442)	0.802		
Nuclear grade (III-IV vs I-II)	4.077 (2.596~6.404)	<0.001		
Necrosis	4.169 (2.668~6.514)	<0.001		
Sarcomatoid differentiation	9.222 (4.561~18.648)	<0.001	4.866 (2.213~10.698)	<0.001
Rheumatoid differentiation	7.646 (3.277~17.839)	<0.001		
PIS				
0	Ref		Ref	
1	2.609 (1.616~4.210)	<0.001	2.577 (1.508~4.403)	0.001
2	5.569 (2.895~10.715)	<0.001	4.087 (1.980~8.438)	<0.001

**Abbreviations:** BMI: Body mass index; AJCC: American Joint Committee on Cancer; CCRCC: Clear cell renal cell carcinoma; PIS score: platelet index score.

**Table 4:** Comparison of our nomogram and other reported models in study cohort

Models	C-index for our study cohort		
	2-year	3-year	4-year
Nomogram	0.902	0.881	0.810
SSIGN	0.876	0.821*	0.775
Leibovich	0.813*	0.764*	0.772
Cindolo	0.798*	0.732*	0.642*
Yaycioglu	0.804*	0.737*	0.648*
MSKCC	0.862*	0.810*	0.761*
Karakiewicz	0.851*	0.793*	0.747*
AJCC stage	0.863*	0.837*	0.759*

**Notes:** *: means the P<0.05 in comparison of C-index between our nomogram and other models.
