# Supplementary material for: Development and validation of nomogram based on a novel platelet index score to predict prognosis in patients with renal cell carcinoma
Source: J Cancer. 2021 Aug 28;12(21):6301–9. doi: 10.7150/jca.60268 (PMC8489122; doi:10.7150/jca.60268)
Supplement: Supplementary file 1 — Supplementary figures. [file jcav12p6301s1.pdf]

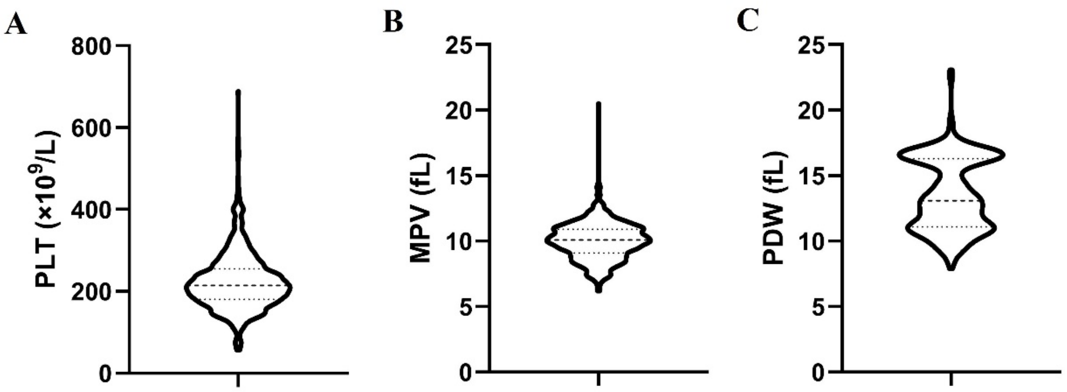

Supplemental 1. The detailed profile of platelet index in our study cohort. (A) PLT (range: 57~689×10^9/L; mean: 223.45±69.31×10^9/L); (B) MPV (range: 6.2~20.5fL; mean: 10.01±1.4fL); (C) PDW (range: 7.9~23.1fL; mean: 13.57±2.78fL).

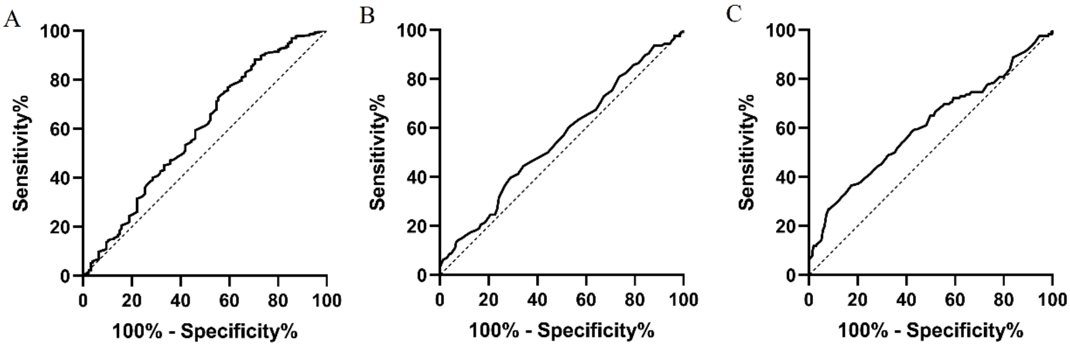

Supplemental 2. Optimal cut-off values were 285×10^9/L, 9.45fL, 10.95fL for PLT, MPV, and PDW respectively. (A) ROC curve for PLT; (B) ROC curve for MPV; (C) ROC curve for PDW.
